# The argument-based approach to validity: theory, assumptions, evidence, and consequences

**DOI:** 10.1007/s11136-026-04194-z

**Published:** 2026-06-06

**Authors:** Melanie Hawkins, Richard Sawatzky, Kevin Weinfurt, Sharla Cartner, Scott Gill, Richard Osborne, Nancy Mayo, Kara Schick-Makaroff, Mirjam Sprangers, R. J. Wirth, Gerald R. Elsworth, Sandra Nolte

**Affiliations:** 1 Global Health and Equity Development Hub, La Trobe University, Melbourne, Australia; 2 School of Nursing, Trinity Western University, Langley, BC Canada; 3 Centre for Advancing Health Outcomes, Providence Health Care Research Institute, Vancouver, BC Canada; 4 Institute of Health and Care Sciences, and Centre for Person-Centered Care (GPCC), Sahlgrenska Academy, University of Gothenburg, Gothenburg, Sweden; 5 Faculty of Nursing, University of Alberta, Edmonton, AB Canada; 6 Center for Health Measurement, Department of Population Health Sciences, Duke University School of Medicine, Durham, NC USA; 7 Research Unit, Justice Health and Forensic Mental Health Network, Sydney, Australia; 8 Department of Medicine, Faculty of Medicine and Health Sciences, McGill University, Montreal, Canada; 9 Department of Medical Psychology, Amsterdam University Medical Centers, University of Amsterdam, Amsterdam, The Netherlands; 10 Vector Psychometric Group, LLC, Chapel Hill, NC USA; 11 Person-Centred Research Directorate, Monash Eastern Health Clinical School, Monash University, Melbourne, Australia

**Keywords:** Validity, Validation, Patient-reported outcomes measures, PRO, Argument-based approach to validity, Health equity

## Abstract

**Background:**

Concerns about measurement validation are often expressed as imperatives to use a “valid measure”. However, validity is not a characteristic of a measure. Instead, validation and validity refer to score interpretation within a context of use. This is important because responses to health measures can be different in different contexts, influencing equitable consequences of measurement. In this paper, we aim to (1) outline the theory of the argument-based approach to validity, and (2) discuss assumptions and evidence in relation to equitable consequences of measurement.

**Methods:**

The argument-based approach to validity asks us to first state how scores will be interpreted and used in context. Assumptions underpinning this statement guide validation planning. Existing and new evidence need to be examined and evaluated in relation to the assumptions and concept of interest, leading to a reasoned evidence-based argument about the degree to which score interpretation in a context of use is valid, with consideration of potential threats to validity and measurement consequences.

**Results:**

Key assumptions are described, including why evidence is needed, what evidence tells us, and the importance of assumptions in relation to the equitable consequences of measurement.

**Conclusion:**

The argument-based approach to validity shifts the focus of validation to a score’s interpretation and use in a context, in relation to the concept of interest. A validity argument is built from evidence about the plausibility of the score interpretation in the context of use with consideration of the degree to which measurement consequences will lead to the intended beneficial health consequences and not perpetuate existing inequities in health.

## Abstract (plain language summary)

**Why is this study needed?** To help healthcare providers with health decisions, patients often fill out questionnaires about their health. Questions may ask how people feel; for example, if they are sad, anxious or stressed. Other questions may ask if people have any pain or other problems with their body, or if they have support from friends and family. When asking these questions, we assume that everyone understands and responds to the questions in the same way. But it is possible that different groups of people interact with the questions in different ways. This means that the data we get should not be interpreted in the same way across these different groups.

**What is the key problem this study addresses?** If different people or groups of people understand and respond to data in different ways, but we interpret that data in the same way, we may make conclusions that lead to unfair decisions about health and health care.

**What is the main point of this study?** To ensure that questions are understood in the same way by different respondents, it is important to do research and ask people from different backgrounds about their understanding of our questionnaires and how to improve them.

**What are the main results and what do they mean?** In this publication, we describe a methodology for exploring questionnaires and the interpretation of self-reported health data. We hope that our research helps to find out if we are asking good questions and if the data we collect and interpret lead to fair decisions in health.

## Introduction

When developing or selecting a measure to collect self-reported data in health contexts, concerns about measurement validation are often expressed as imperatives to use a “valid measure”. However, it is important that we consider what the term “valid measure” implies. The often-unquestioned assumption underlying validity is that the scores will have the same meaning and consequences in different contexts of use. Indeed, assumptions are made that the measure functions the same way across different groups of people, different contexts of use, and even, to some degree, for different concepts (or constructs) of interest (i.e., what is being measured). Yet people in diverse populations have different backgrounds and experiences that influence how they interpret and respond to questions that are used to measure the concept of interest (e.g., patient-reported outcomes (PROs), including health and quality of life) [1, 2]. When this is ignored, the measurement can hide or misrepresent the concept of interest for some groups of people, which can lead to harmful health consequences and exacerbation of health inequities (e.g., due to health concerns not being detected for some groups of people). Prominent validity theorists have long recognised that these interpretations and response differences can mean that the consequences of measuring these outcomes may put certain persons or groups of people at a disadvantage [3–9]. Validity thinking has thus shifted from the binary notion of a valid/not valid measure to one focused on evaluating evidence for and against key assumptions underpinning score interpretation within a specific context [10]. That is, validity is not expressed through a fixed property of a measure, but rather through an evaluative argument about inferences derived from data obtained via the measure. This paper describes a theory-driven methodology for exploring questionnaires and interpreting self-reported health data: the argument-based approach to validity [5, 7, 11].

Since the early 20th Century, validity theorists have been continually progressing approaches to measurement validation [3, 5, 7, 12–24]. Collectively, this work informed the authoritative validation reference, the 2014 Standards for Educational and Psychological Testing (the Standards) [25]. The Standards was written by a joint committee of measurement specialists and provides consensus definitions of validation and validity. More specifically, the Standards define validation as “…a process of constructing and evaluating arguments for and against the intended interpretation of test scores and their relevance to the proposed use” (p.11) and validity as “…the degree to which evidence and theory support the interpretations of test scores for proposed uses of tests” (p.11). These definitions emphasise that validation is a *process of investigation* about score interpretation and use in context, and validity is about the *evaluative judgment or validity argument*, based on the results of the investigation [3, 5, 26–31]. Here, the word “argument” refers to an evidence-based reasoned logic that justifies a score interpretation in a specific context of use. The methodological approach to validation planning described in this paper is grounded in the argument-based approach to validation [5, 7, 11, 22, 23].

Whilst drawing on the history of validity theory discourse, Kane’s argument-based approach to the validation process is theoretically founded on Stephen Toulmin’s model of practical argument, also called practical reasoning. Toulmin’s model has a focus on evaluating inferences, or claims, made (e.g., in this context, claims made about score interpretation) [32, 33]. Kane’s central premise is that validity is about evaluating a contextual interpretation-use argument, not evaluating a measurement instrument itself; that validity is not about seeking truth, but about evaluating the *plausibility* of an interpretation-use argument in a context, based on empirical evidence and a clearly defined concept of interest [34]. For example, quality of life as a PRO is a concept of interest that is defined by context and purpose, and it needs to be measured accordingly [35]; therefore, decision making relies on the evidence-based plausibility of score interpretation in that context. The argument-based model pivots validation research in health from checklist-driven psychometrics to a contextualised reasoning process that ties sources of evidence to a validity claim about score inferences (e.g., inferences about whether a PRO has improved or not, or group differences in PROs). Existing evidence frameworks for collecting validity evidence are relevant and useful, but Kane’s model provides a theoretical scaffolding for organising validity evidence into transparent coherent arguments regarding the degree of confidence about how and why the evidence supports score interpretation in a context of use in relation to a concept of interest.

This paper adopts a contemporary unified view of validity as articulated by Messick [3], which was further operationalised in Kane’s argument-based approach to validity [5, 7, 11], and which we now apply to measurement in health. For the purposes of this paper, we use the term “concept of interest” and “construct” as mostly synonymous. The most recent U.S Food and Drug Administration (FDA) Patient-Focused Drug Development (PFDD) guidance documents are informed by modern validity theory (specifically, Kane’s argument-based approach) and use the term “concept of interest” (i.e., what is being measured) [30]. We acknowledge and recognise the historical origin, purpose and importance of the technical term “construct” in the psychological measurement literature [3, 24, 36]. A useful modern definition of construct is provided by Hubley and Zumbo: “A *construct* may be conceived of as a concept or mental representation of shared attributes or characteristics, and it is assumed to exist because it gives rise to observable or measurable phenomena” [37]. Our paper seeks to provide practical validity testing methodology, with examples of methods, that can be useful across a wide range of health research.

The argument-based approach to evaluating the validity of score-based inferences incorporates the need to explicitly state, at the very beginning of the validation process, the concept of interest, context of use, and, importantly, the proposed score interpretation (e.g., proposed interpretations about what PRO scores, including higher and lower scores, will mean for a person or group of people in relation to the concept of interest). The approach can be likened to testing a scientific hypothesis and the assumptions underpinning it through evidential investigation [7]. The following steps enable the development of an evidence-based reasoned logical argument about score interpretation in a context of use:

1. Define the concept of interest
2. Define the context of use
3. Define how a score will be interpreted
4. Outline key assumptions underlying this interpretation
5. Accumulate evidence supporting/contradicting each assumption
6. Evaluate which, if any, key assumptions require further investigation and plan investigations into these assumptions
7. Conduct planned investigations
8. Repeat #6 & #7 as needed

It is worth noting that the argument-based approach does not change the study designs or the qualitative and quantitative validity testing methods we already use. But it does *shift our thinking* about the meaning and consequences of a declaration of validity. If validity is thought of as a property of a measure, then the danger is to think that interpretation of the scores will have the same meaning in any context and, thus, the same consequences. But when validity refers to score interpretation and use in a specific context, then it is not reasonable to assume that score-based interpretations will yield the same meaning and consequences for other uses and in other contexts [38]. We need to be aware of the assumptions we (often unconsciously) make when we measure health. In this paper, we aim to (1) outline the theory on which the argument-based approach to validity is based, and (2) discuss assumptions and validity evidence in relation to equitable consequences of measurement.

## Theory

The Standards is the latest edition of an established body of work around validity theory in educational and psychological testing that goes back to its initial publication more than 50 years ago, followed by various iterations [39–43]. Each iteration records the consensus thinking of its time about validity and validation. Note that a Joint Committee was established in June 2024 and is currently working on a revision of the 2014 Standards, which emphasises the continuous evolution of this field of research [44].

In the 1974 Standards, validation practice was operationalised through reporting types of validity with the focus on quantitative studies of a measure’s psychometric properties [41]. It is this version of the Standards that influenced and guided the development of validation practice in self-reported health measurement [45]: e.g., PRO measures and other self-report health measures. In the mid-1980s, the unified theory of validity united the different types of validity under recommendations to demonstrate an integrated evaluation of evidence about a proposed score interpretation for a specified use and context, as based on the concept of interest [3, 46, 47]. The 1985, 1999 and 2014 Standards iteratively incorporated clearer and stronger recommendations that validity is about score interpretations for specific uses in specific contexts. Importantly, the unified theory of validity of Samuel Messick drew attention to social and ethical values and the consequences of measurement [4, 47–50]. However, after adoption of the 1974 Standards, validity developments in health measurement remained largely separate from the subsequent progress in validation theory and practice that was made in education and psychology.

The unified concept of validity progressed the practice of measurement validation from a focus largely on psychometric properties to a focus on thinking about the meaning of scores (i.e., score interpretation) for different uses (i.e., the purpose to which scores will be put) in different contexts (e.g., population, language, culture, socio-economic). Validation became “an integrated evaluative judgement of the degree to which empirical evidence and theoretical rationales support the *adequacy* and *appropriateness* of *inferences* and *actions* based on test scores” (Messick’s italics) [3]. Important advancements in validation practice include the incorporation of the value implications, including fairness, of using score-based inferences to make decisions that have social consequences, and this emphasis on consequences rippled through validity thinking [8, 31, 47, 51–56].

The integration of validity theory from (mainly) education into health measurement practices began with seminal papers from health education, especially by Zumbo and colleagues [8, 31, 54, 57, 58], which were followed by others interested in measurement in health [27–29, 59–63], including the incorporation of the argument-based approach to validity into the most recent PFDD guidance documents published by the FDA [64], in particular Guidance 3 [30]. Through the methodology of the argument-based approach (which the FDA calls the rationale-based approach), practitioners in self-reported health measurement gain access to decades of thinking about evaluating the validity of inferences derived from scores. While Kane’s model cautiously incorporates consequences into the evaluation of score interpretation and use, especially for high stakes decisions, the argument-based model described in this paper is influenced by the thinking of Messick, as well as Zumbo and colleagues, about intended and unintended consequences of measurement being sources of validity evidence [3, 47–50]. Such thinking involves considering validity evidence based on intended personal and social consequences [8, 37, 54], especially the extent to which measurement leads to equitable decisions and actions, as well as evidence of unintended side effects, including negative or inequitable consequences [3, 5, 7, 8, 11, 31, 54, 65]. Developments by Zumbo and colleagues extend Messick’s theoretical developments to incorporate essential matters of measurement invariance that support or threaten measurement claims within measurement contexts [8, 54].

## Assumptions

When we measure, we make assumptions, whether we are aware of them or not. For example, we assume that people interpret and respond to items in ways the measure developers intended; we assume the concept we are measuring means the same thing to new respondents as for previous respondents; and we assume the outcomes of measurement will bring some benefit to someone (i.e., patients, communities, populations, organisations, governments). Measurements of health outcomes are interpreted and used in many different contexts by clinicians [66, 67], health organisations [68, 69], and regulatory bodies [30, 70] to make decisions about people’s health care and medical treatments. The assumptions underpinning a score interpretation in a context of use must be supported by evidence to ensure that subsequent actions do not reinforce or introduce health inequities, especially for populations already experiencing disadvantage [71, 72]. Unexamined assumptions carry the danger of misinterpreting scores, potentially resulting in unfair, inequitable and possibly harmful health consequences. Importantly, a validation process should evaluate the extent to which score-based decisions may misrepresent people, perpetuate measurement biases, exacerbate inequities, or lead to poor decisions about health treatments, interventions, and policies [73]. We present examples of key assumptions underpinning score interpretations in the first column of Table 1, with rationale and evidence for each assumption in columns 2 and 3. Many of these assumptions are likely to be familiar to the readers of this article, but the steps of the argument-based approach make them explicit and observable, so we can logically evaluate them [59]. Table 1 is not a checklist, but an example of assumptions that comprise an integrated chain of critical reasoning about score interpretation in a context of use. There may be other important assumptions, depending on the measure being used and the proposed score interpretation, use and context. Also note that not all these assumptions will always be relevant to a validity argument. Only assumptions relevant to score interpretation in a context of use need to be investigated, and some assumptions will require more or stronger evidence than others. What is important in Table 1 is that underpinning Kane’s argument-based model is a process of critical reasoning about what validity evidence is needed and why. Such reasoning includes identifying assumptions that may threaten the validity of score inferences. The resulting interpretation-use arguments form the basis for developing a validity testing plan, as exemplified in the companion paper about evaluating score-based inferences derived from a health literacy self-report measure for use in prisons in New South Wales, Australia (10.1007/s11136-026-04269-x).

**Table 1:** Key assumptions underpinning score interpretations^1^, why evidence is needed, and what the evidence tells us about the assumption

Key assumptions about…	Why evidence is needed	What the evidence tells us about the assumption^2^
**…the content of a measure**		
The content of a self-report measure in health captures the important and relevant aspects of the concept of interest according to the proposed interpretation in the context of use, including content related to the self-report measure’s themes, wording, format of items, administration, and scoring **Note**: The evidence focus for this assumption is on the applicability of the content for measuring the concept of interest	Concepts of interest are influenced by context and an appropriate definition of content in one context may not be applicable in another The content of the measure needs to represent the concept of interest as perceived by the target population in the context of use Different formats of data collection are more and less appropriate for different groups of people in different contexts When using a measure for a new purpose or new (or across) contexts of use, content must be assessed for construct underrepresentation^3^ and construct-irrelevant variance^4^ in relation to the concept of interest and the target population	Ideally,… The content of the items meaningfully represents the target population’s experiences of the concept of interest The measure’s instructions and format are understood by the target population The process of administering the measure is appropriate for the target population and correctly applied The scoring procedure is appropriate for the target population and correctly applied There is absence of threats to the validity of score interpretation in the context of use, based on the concept of interest, content of the measure, and the target population
**…how people think about and respond to the content**		
The target population’s response processes (i.e., understanding, interpreting, thinking about, feelings about, and responding) to the measure’s content (i.e., the items) are as intended by the measure developer(s). The target population includes respondents (e.g., patients, community members) as well as those using the score interpretations to make decisions about health (i.e., clinicians, researchers, policy makers) **Note**: The evidence focus for this assumption is on the degree to which people’s responses to the items are aligned with the intent of the items	Concepts of interest are perceived differently in different contexts, which means the ways in which respondents engage with items can vary across contexts People’s interpretations and responses to items need to align with item intents and be consistent between respondents, and between respondents and users of scores, for valid score interpretation in each context of use	Ideally,… Respondents perceive the concept of interest (as measured through item content) as intended by the measure developers Respondents from the target population understand, interpret, cognitively assess (think and feel about), and respond to items as intended Those using score interpretations for decision making understand, interpret, think and feel about the items, and interpret responses from the target population, as intended by the measure developers
**…how the scoring works**		
The method of scoring responses (e.g., response options, scaling) is appropriate for assessing the concept of interest **Note**: The evidence focus for this assumption is on determining the appropriateness of the method for respondents to provide their responses to items	Response options and scaling must correspond to and accurately reflect the concept of interest, as indicated by the items The method of scoring responses needs to function reliably, accurately, and equitably (i.e., fairly) across respondents	Ideally,… Respondents in the target population can reliably, accurately and equitably provide their responses to items in ways that reflect their diverse experiences of the concept of interest
**…how scores relate to real-world experiences**		
Scores correspond to people’s lived experiences that are related to the concept of interest **Note**: The evidence focus for this assumption is on score meaning for decision making in the real-world	Concepts of interest may be perceived differently in different contexts. To make decisions based on self-reported health outcomes (e.g., to inform and justify health interventions, practice and policy), score interpretations must appropriately, meaningfully and accurately reflect the concept-related lived experiences of the target population in the context of use	Ideally,… The scores reflect diverse real-world experiences and correspond meaningfully to the concept of interest as hypothesised, indicating the concept has concrete meaning for the target population Item content accurately represents concept-relevant experiences and understandings of diverse individuals in the target population
**…how the items work with each other**		
Item scores fit the hypothesised structure of the measure **Note**: The evidence focus for this assumption is on the degree to which the relationships among items, as assessed through item scores, conform to the concept of interest	The internal structure of the relationships among the items needs to be consistent with the theoretical view of the concept of interest to ensure the same concept is being measured in the same way in each population and context of score interpretation and use Concepts are context dependent, and a measure’s internal structure needs to be acceptably replicated in new populations and contexts	Ideally,… Item scores in the target population indicate that the measure’s internal structure, as theorised by the concept of interest, is acceptably replicated
**…potential risks to score interpretation and use in context**		
Scores are not unduly influenced by factors that are unrelated to the concept of interest (i.e., there are no threats to validity due to construct-irrelevant variance) **Note**: The evidence focus for this assumption is on the potential for unintended negative consequences to threaten the validity of the intended score interpretation in the context of use	Scores will not accurately reflect the concept of interest if they are influenced unduly by construct-irrelevant factors, particularly if those factors operate differently for different respondents and/or across different contexts	Ideally,… The variance among scores is shown to be clearly and predominantly influenced by variation in the concept of interest Construct-irrelevant variance is shown to be minimal
**…score change over time**		
Scores are sufficiently sensitive to detect meaningful differences within individuals and between groups of individuals over time, in expected directions and as hypothesised by the theory of the concept of interest **Note**: The evidence focus for this assumption is on meaningful change over time within and between individuals of the target population	Self-report scores need to accurately reflect theorised and meaningful change over time within individual people and between groups of people to support equitable real-world decisions in a timely manner (e.g., tracking patient progress, evaluation of interventions)	Ideally,… Scores are influenced by experimental conditions or temporal effects (time periods) in expected directions Sensitivity to changes in scores for individuals and between groups of individuals in the target population reflect hypothesised changes related to the concept of interest
**…the way scores relate to other variables**		
The scores correlate as expected to scores on criterion measures (e.g., other measures of the same concept of interest) or other external variables (e.g., sociodemographic variables) **Note**: The evidence focus for this assumption is on how the concept of interest is related to other variables as indicated by respondents’ scores	To demonstrate the meaning and distinctiveness of the concept of interest, self-report scores must be associated as expected with the scores of other measures that are based on conceptually related and unrelated concepts Scores must be associated as expected with other variables, such as sociodemographic data, to detect group differences as hypothesised by the concept of interest	Ideally,… Scores demonstrate stronger associations with convergent constructs (concepts of interest) and weaker associations with divergent constructs in patterns that are in accordance with the theory of the concept of interest Scores demonstrate the expected correlations with other variables, as hypothesised
**…the consequences of measurement**		
As related to issues of validity, the consequences of score interpretations lead to the intended benefits and minimise unintended consequences, especially those that may do harm **Note**: The evidence focus for this assumption is on the realisation of expected beneficial consequences and the extent to which negative consequences are minimised	Information from measurement outcomes informs a wide range of decisions about people’s health so the consequences of measurement must be more beneficial than harmful for the target population As related to the validity of score interpretation in a context of use, measurement outcomes should deliver the expected benefits to the target population Measurement outcomes must not misrepresent people, perpetuate measurement biases or exacerbate inequities	Ideally,… Measurement processes are fair and equitable Measurement outcomes support clear and justified decision making that leads to the intended beneficial changes for the target population The measurement outcomes achieve the intended benefits and minimise unintended negative consequences in the target population There is no evidence of measurement outcomes leading to misrepresentation, measurement biases, or inequitable consequences

Adapted from Table 2 (p.19) [30]

^1^Scores obtained from self-report measures in health

^2^This is under ideal circumstances. Caveats may be needed

^3^Construct underrepresentation refers to the degree to which a measure fails to capture important aspects of a construct in a context of score interpretation and use.

^4^Construct-irrelevant variance refers to the degree to which scores are influenced by factors that are unrelated to the construct being measured in a context of score interpretation and use

## Validity evidence

The type, strength and quantity of validity evidence required is dependent on the extent to which an assumption may influence the consequences of measurement. That is, if decisions based on measurement are likely to have a profound impact on a target population (e.g., people receiving treatment for complex mental health conditions), then more or a wider range of evidence may be needed to increase confidence in the score interpretation [6, 25].

Despite the many investigative methods used in validation studies, validity evidence is generally based on five sources: (1) the content of the measure, (2) the response processes of respondents and users, (3) the internal structure of the measure, (4) the relationships between scores and other variables, and (5) the consequences of measurement as related to validity considerations [25]. Different assumptions require different types of evidence, and thus different methods of investigation need to be applied. While validation practices in health have relied mostly on methods that produce quantitative psychometric evidence, there is now recognition of the importance of the use of qualitative and mixed methods approaches to generate validity evidence. Research now demonstrates the need for evidence about the ways in which people interact with, think and feel about, and respond to items – that is, the often-under-reported response processes evidence [28, 74–76].

The argument-based approach to validity shifts the focus of investigation from the traditional reporting of primarily psychometric properties to an integrated evaluation of a range of qualitative and quantitative evidence about key assumptions. However, importantly, it does not change the investigation methods we know and use. To help resolve concerns about the differences between assessing validity through types of evidence versus the more commonly known “types-of-validity” approach, we present Table 2. Conventional measurement validity procedures and criteria provide a structured categorisation of empirical evidence. The argument-based approach has a focus on why and how different sources of empirical validity evidence are needed and used to evaluate interpretive claims (see also 10.1007/s11136-026-04269-x).

**Table 2:** Types of validity evidence, types of validity from conventional validity frameworks, and investigation method examples

Types of validity evidence^1^	Types of validity from conventional validity frameworks	Examples of methods for these investigations
1. Content evidence	Content validity	Expert review, literature review
2. Response processes evidence	No equivalent	Cognitive or think-aloud interviews, focus groups, observations in clinical settings, response time analyses, eye tracking, records that monitor the development of a response, relationships among test components or between test scores and external variables, paradata (e.g., mouse clicks, accessing definitions of terms or explanations with examples, changing responses), statistical or computational modelling of response behaviour
3. Internal structure evidence	“Construct validity”^2^	Factor structure (e.g., categorical confirmatory factor analysis, item response theory)
4. Relations to external variables evidence	Criterion validity “Construct validity”^2^	Known-groups analysis, correlation analyses
5. Consequences of measurement evidence	No equivalent	Process and outcomes evaluation, meta-analyses

Sources of evidence in the first column are from the 2014 Standards [25]. Open access files: https://www.testingstandards.net/open-access-files.html

^1^While reliability evidence is an important source of evidence about the consistency of a measurement, it is not validity evidence and so is not included in this table [25]

^2^ “Construct validity” is a catch-all term that does not clearly describe the validity evidence being presented. As described by Cronbach: “The great run of test developers have treated construct validity as a wastebasket category. In a test manual, the section with that heading is likely to be an unordered array of correlations with miscellaneous other tests and demographic variables. Some of these facts bear on construct validity, but a coordinated argument is missing.”[77]

## Consequences

Validity testing studies often report content evidence, internal structure evidence, and evidence about score relationships with other variables (these are often, and as we argue in this paper, rather imprecisely, reported as specific “types of validity”). However, evidence based on response processes and consequences of measurement is usually lacking from validity studies in health [75, 78]. Validation standards and guidelines also frequently omit these two very important types of evidence [79]. Let’s look at the importance of these two sources of validity evidence.

*Response processes* evidence can be confused with evidence generated about the content of a measure, but these are different. To understand the response processes of people appraising and engaging with the content of a self-report measure, we need insights into what people do, think, and feel when cognitively interacting with, thinking about, and formulating responses to items, while remembering related information and experiences [80–82]. There is a stark absence of response processes evidence in the validity testing literature in health, yet this source of evidence is so important for revealing biases that cause distorted score meaning for some people in some contexts and for some score uses, thereby potentially leading to inequitable decision making [31, 75, 83]. In the absence of supportive evidence, and prior to large quantitative studies, investigation should occur into respondents’ cognitive (response) processes and the extent to which scores represent their experiences related to the concept of interest. These investigations are especially needed when applying score interpretations in new uses and contexts. Such investigations can be done by comparing respondents’ narratives about each item and score with descriptions about the intent of each item, sometimes called concept definitions [84], as defined by the authors of the measure [76, 85].

The beneficial *consequences* arising from what scores mean in one context may not transfer to health and equity benefits in another context (e.g., a different cultural context) [28, 86, 87]. Unintended negative consequences of measurement can result from invalid or wrong interpretation and use of scores in a context, based on incorrect or insufficiently investigated assumptions. The intended consequences of self-reported health measurement are, broadly speaking, to understand and improve health outcomes and reduce inequity, but unintended consequences – perhaps due to contextual differences – can lead to entrenchment or even (unintentional) promotion of health inequities [71, 73].

A score interpretation in a context of use is proposed prior to or in conjunction with validation planning. Identified assumptions drive the development of the validation plan and may identify potential threats to the validity of the proposed score interpretation and context of use. The investigation of the assumptions aims to determine the extent to which those threats could lead to unintended consequences of score interpretation in the context of use. There are two main types of threats to validity [3]: construct underrepresentation and construct-irrelevant variance.

The first threat, *construct underrepresentation*, is when important aspects of a construct (i.e., concept of interest) are not sufficiently captured by a selected measure. Construct underrepresentation can occur, for example, when a measure from one context (e.g., the measure development setting) is used in another context (e.g., a different country or cultural group) without checking the construct meaning (i.e., its conceptualization) in the new context (e.g., without response processes evidence). Lack of recognition of, and assumptions about, the knowledge and experience that people and communities hold about their own health can lead to epistemic injustice and bias in health measurement [71, 88–90]. Inadequate engagement with patients, health workers, and other community members during the development, selection or adaptation of a measure can result in the measurement of constructs that do not adequately or appropriately represent the perspectives or experiences of the study population [71, 91].

The second threat, *construct-irrelevant variance*, is when factors external to the concept of interest affect individuals’ responses to the items. When this occurs, the scores will not have the same meaning for different people, which may lead to misinterpretation of scores and wrong decisions based on those interpretations. External factors may be situational, cultural, ecological or other social, economic, or political contexts that influence people’s lives, their health experiences, and therefore the ways in which they respond to items [31]. Investigation into response processes helps to identify factors leading to variance in responses in different populations. Response process evidence determines the extent to which respondents engage as intended with the concept of interest and can help detect extraneous factors that influence that engagement [75, 92].

## Conclusions

The argument-based approach to validity helps us to plan and carry out our validation work with integrity to ensure we undertake relevant investigations of key assumptions that may affect how health outcomes scores will be interpreted and used in different contexts. A validation plan supports consideration of not only validity threats, but also intended and potential unintended consequences of measurement. With this approach, qualitative validity evidence (e.g., evidence related to content and response processes) is just as crucial as quantitative (psychometric) evidence for understanding the meaning and relevance of score interpretations in a context of use. Qualitative evidence is often needed in new contexts where it is important to explore differences in the ways in which respondents conceptualize, engage with, interpret, think about, and respond to items. These investigations are important because consequences arising from measurement biases can lead to inequitable decisions about health and health care. The importance of the argument-based approach to validity is that it embraces equity implications related to decisions that have health and social consequences [31, 54, 56, 71].

As mentioned earlier in this article, the FDA has released its updated PFDD guidance series for the use of PRO and other clinical outcome assessment (COA) measures in drug development [64]. In particular, the 2025 Guidance 3 (Patient Focused Drug Development: Selecting, Developing, or Modifying Fit-for-Purpose Clinical Outcome Assessments) is influenced by the argument-based approach to validity. Guidance 3 notes that decisions about whether a measure is deemed “fit-for-purpose” are based on how clearly the intended concept of interest and context of use are described and the sufficiency of evidence “to support a clear rationale [i.e., argument] for the proposed interpretation and use” (p.9) of the measure [30]. Also, the International Council for Harmonisation of Technical Requirements for Pharmaceuticals for Human Use (ICH) argued in their 2021 PFDD reflection paper that "Whether a COA is fit-for-purpose is determined by the strength of the evidence in support of interpreting the COA scores as reflecting the concept of interest within the context of use" [93]. Hence, the argument-based approach to validity is slowly but surely infiltrating regulatory agencies’ evaluation of the adequacy of selected health outcomes measures in view of available evidence supporting the interpretation of scores in a context of use.

Self-reported measurement of health outcomes is a contextualised practice. People’s responses to health outcome measures are influenced by the places and situations in which they manage their health: i.e., where they are born, grow, live, work and age [94]. Scores collected from respondents are not just numbers. These scores tell us stories about real people with real health issues in real lives. We need to be confident about the extent to which the concepts we measure have meaning for respondents and that the inferences we derive from scores reflect their lived experiences. The argument-based approach to validity requires no new methods and only a shift in perspective – from thinking of validity as being the property of a measure to validity being about score interpretation in a context of use and in relation to a concept of interest. The validation process is informed by key assumptions, which guide the collection of existing and/or generation of new relevant types of validity evidence, with consideration of the equitable consequences of measurement. The argument-based approach to validity helps us to determine the extent to which we can have confidence that our measurement of health will not do harm or perpetuate existing inequities in health and will lead to the intended beneficial health consequences.
